# Zero-Dose Childhood Vaccination Status in Rural Democratic Republic of Congo: Quantifying the Relative Impact of Geographic Accessibility and Attitudes toward Vaccination

**DOI:** 10.3390/vaccines12060617

**Published:** 2024-06-04

**Authors:** Branly Kilola Mbunga, Patrick Y. Liu, Freddy Bangelesa, Eric Mafuta, Nkamba Mukadi Dalau, Landry Egbende, Nicole A. Hoff, Jean Bosco Kasonga, Aimée Lulebo, Deogratias Manirakiza, Adèle Mudipanu, Nono Mvuama, Paul Ouma, Kerry Wong, Paul Lusamba, Roy Burstein

**Affiliations:** 1 Kinshasa School of Public Health, University of Kinshasa, Kinshasa H8Q3+2HV, Democratic Republic of the Congo; branly.mbunga@unikin.ac.cd (B.K.M.); ir.fbangelesa@hotmail.fr (F.B.); eric.mafuta@unikin.ac.cd (E.M.); dalau.nkamba@unikin.ac.cd (N.M.D.); landry.egbende@unikin.ac.cd (L.E.); jeanbosco.kasonga@unikin.ac.cd (J.B.K.); aimee.lulebo@ksph-lisanga.org (A.L.); nono.mvuama@unikin.ac.cd (N.M.); paul.lusamba@unikin.ac.cd (P.L.); 2 Health and Life Sciences, Gates Ventures, Seattle, WA 98033, USA; patrick.liu@gatesventures.com; 3 Institute of Geography and Geology, University of Würzburg, Am Hubland, 97074 Würzburg, Germany; 4 Fielding School of Public Health, University of California, Los Angeles, CA 90095, USA; nhoff84@ucla.edu; 5 United Nations Children’s Fund (UNICEF) Country Office, Kinshasa M7H9+HQW, Democratic Republic of the Congo; dmanirakiza@unicef.org (D.M.); amudipanu@unicef.org (A.M.); 6 World Health Organization, 1211 Geneva, Switzerland; oumap@who.int (P.O.); wongk@who.int (K.W.); 7 Bill & Melinda Gates Foundation, Seattle, WA 98109, USA

**Keywords:** zero-dose, children, vaccination, immunization, geographic accessibility, vaccine demand

## Abstract

Despite efforts to increase childhood vaccination coverage in the Democratic Republic of the Congo (DRC), approximately 20% of infants have not started their routine immunization schedule (zero-dose). The present study aims to evaluate the relative influence of geospatial access to health facilities and caregiver perceptions of vaccines on the vaccination status of children in rural DRC. Pooled data from two consecutive nationwide immunization surveys conducted in 2022 and 2023 were used. Geographic accessibility was assessed based on travel time from households to their nearest health facility using the AccessMod 5 model. Caregiver attitudes to vaccination were assessed using the survey question “How good do you think vaccines are for your child?” We used logistic regression to assess the relationship between geographic accessibility, caregiver attitudes toward vaccination, and their child’s vaccination status. Geographic accessibility to health facilities was high in rural DRC, with 88% of the population living within an hour’s walk to a health facility. Responding that vaccines are “Bad, Very Bad, or Don’t Know” relative to “Very Good” for children was associated with a many-fold increased odds of a zero-dose status (ORs 69.3 [95%CI: 63.4–75.8]) compared to the odds for those living 60+ min from a health facility, relative to <5 min (1.3 [95%CI: 1.1–1.4]). Similar proportions of the population fell into these two at-risk categories. We did not find evidence of an interaction between caregiver attitude toward vaccination and travel time to care. While geographic access to health facilities is crucial, caregiver demand appears to be a more important driver in improving vaccination rates in rural DRC.

## 1. Introduction

Immunization is widely regarded as one of the most important public health interventions for reducing childhood morbidity and mortality. Despite significant progress in global coverage over the past two decades, substantial inequities persist [1,2,3]. Of the 20.5 million under-immunized (UI) children in 2022, 70% or 14.3 million were considered zero-dose (ZD), operationally defined as children who have not had a single dose of the Pentavalent vaccine [4]. Global efforts, including the Immunization Agenda 2030 (IA2030), have recognized the imperative of reaching zero-dose children as a strategic priority, calling for a 50% reduction in the number of zero-dose children by the year 2030 [5,6]. Beyond the risk of vaccine-preventable diseases, children who are ZD or UI and their families face multiple deprivations related to poverty, gender inequities, and access to other health systems services beyond routine immunization [3,7,8,9].

The Democratic Republic of the Congo (DRC) persists as one of the most vulnerable countries to vaccine-preventable diseases and has experienced several recent outbreaks, including a measles epidemic in 2019 resulting in more than 300,000 suspected cases and 6000 deaths [10,11]. Prior efforts to understand the drivers of persistently low coverage in DRC identified several system-side challenges, including inequities in service and vaccine availability, variable management and program monitoring, and challenges with health worker motivation [11]. In response, the Ministry of Health developed the Mashako Plan in 2018 with the primary aim of improving coverage interventions focused on improved coordination, service delivery, vaccine availability, real-time service delivery monitoring, and more robust program evaluation. Despite improvements in system targets and increases in vaccination coverage, an estimated 19.1% of children 12–23 months remain ZD (771,000), and 25.5% remain under-immunized (1.03 million) in 2021 [12].

There is a need to move beyond the sole focus on supply-side drivers to more effectively and sustainably overcome the intersecting barriers to vaccination that children who are ZD and UI and their families face [13]. Frameworks like the Behavioral and Social Drivers (BeSD) Framework, UNICEF Journey to Health and Immunization Framework, and Exemplars in Global Health (EGH) Vaccine Delivery Framework identify factors related to vaccine demand, access to services, and supply-side readiness as key drivers of vaccination—and thus barriers to ZD and UI [14,15,16]. Data across these three domains have been limited to date but are becoming increasingly available to assess their impact on vaccination coverage.

There is a growing body of evidence demonstrating the impact of geospatial access to vaccination services and coverage in sub-Saharan Africa, though this has not yet been characterized in DRC [17,18,19,20,21]. Moreover, recent analyses on self-reported reasons for non-vaccination in DRC identified that 82% of caregivers of children who were ZD expressed no intent to vaccinate their child and that 9.6% experienced barriers related to access to services [22]. While this evidence on intent and access adds nuance to the understanding of drivers of non- and under-vaccination in DRC, this insight is limited when they are evaluated in isolation. A more complete understanding of the drivers of coverage requires analyzing their joint influence related to the individual within the broader health system context [13]. What is the relative impact of geospatial access and caregiver intent on ZD status in DRC, and to what extent do they interact? This understanding is needed to better target and prioritize interventions to overcome barriers to vaccination for children who are ZD.

This current study leverages data from the 2021 and 2022 rounds of the Enquête de Couverture Vaccinale household coverage surveys conducted by the Kinshasa School of Public Health (ECV; vaccine coverage survey; fielded in early 2022 and early 2023, respectively) to assess the relative association between the EGH Vaccine Delivery Framework domains of intent to vaccinate (proxied by caregiver attitudes towards vaccination) and geographic access to services on vaccination status among rural households in DRC [12]. We first linked a constructed geolocated health facility list to estimate travel time from household locations to their closest health facility. We then compared the relative association between the estimated travel time and caregivers’ self-reported perceptions of the importance of vaccination on whether their child was ZD, UI, or fully-immunized. Findings from this study may provide important insight relevant to improving targeted programming and policies to address barriers to vaccination in DRC and improve more equitable coverage.

## 2. Materials and Methods

### 2.1. Data Source

This is a secondary analysis of pooled data from two consecutive nationwide immunization surveys (Enquête de Couverture Vaccinale (ECV)), conducted in early 2022 and early 2023 and referred to as ECV2021 and ECV2022, respectively. Additional information on sampling protocols for these surveys can be found in the Supplementary Materials and have been described elsewhere [22,23]. Briefly, the ECVs are nationally representative multi-stage cluster cross-sectional randomized surveys, following a modified version of the WHO sampling method for vaccine coverage, targeting a sample of 6–23-month-olds [24]. GPS coordinates were recorded for each household in both study years. Data were pooled across the two surveys for this study.

### 2.2. Data Preparation

The study sample consisted of children aged 12–23 months and their caregivers. Following the approach used by Gavi, we considered a child to be zero-dose if they did not receive their first dose of the Pentavalent vaccine (DTP-Hib-HepB) by age 12 months [9]. We define under-immunized as receiving the first dose of Pentavalent but not the third or the first dose of the measles-containing vaccine. Given the timing and age cohorts targeted in the two surveys and the randomized sampling, it is extremely unlikely for the same child to appear twice in the two-survey sample.

Our study focused on rural households, defined by the survey’s sampling frame. While travel time may be a concern in urban areas, particularly due to traffic congestion, we focused on rural households for this study because our methods for quantifying it do not incorporate traffic and other urban-specific issues and thus would likely underestimate travel time for urban areas relative to rural ones, where travel time is more a function of the distance, ground cover, and road type.

GPS coordinates from the ECV surveys were used to extract travel time to the nearest health facility for each household. Households with missing GPS coordinates or with coordinates not aligned to the AccessMod surface (see below) were dropped. Some households exhibited duplicated GPS coordinates and were consequently excluded from the analysis after being cross-examined against dataset elements, such as health zones, health areas, village names, or household identifiers. This duplication accounted for 12% of the entire dataset. This did not have an appreciable impact on the main results (see sensitivity analysis described in Supplementary Table S3). Further studies are underway to investigate the causes of this duplication and remedy them for future data collection.

Caregiver attitude toward vaccination was reflected using the survey question “How good do you think vaccines are for your child?” (originally surveyed in French as “*Dans quelle mesure pensez-vous que les vaccins sont bons pour votre enfant?*”) Responses were grouped as “Very Bad, Bad, Don’t Know”, “Good”, and “Very Good”. We combined the “Very bad,” “Bad”, and “Don’t Know” groupings because each had a relatively small sample and each had similar correlations with the outcome. This was one of several questions included in the ECV survey corresponding to well-validated constructs used by the Vaccine Confidence Project and the WHO Behavioral and Social Drivers (BeSD) Tool around vaccine attitudes, including attitudes around vaccine safety, efficacy, peer norms, and more. We selected this single measure to reflect vaccine attitudes because it was highly correlated with the other measures for this sample (Spearman’s rank correlation coefficient of 0.53–0.68) and to simplify the interpretation of the primary analysis comparing the effect of vaccine attitudes and geospatial access on vaccination status.

Maternal age, education level of the household head, and birth order were also retained as control variables.

### 2.3. Quantifying Geographic Accessibility

Health facility locations were derived using a combination of data sources with facility GPS coordinates, including DHIS2 (N = 21,712 facilities, 72% with GPS), monthly Mashako Plan supervision data (N = 15,565 facilities, 92.5% with GPS) (13), and the ECV facility survey conducted alongside the ECV household survey (N = 2904 facilities, 100% with GPS). We used the DHIS2 as a reference master facility list and supplemented the GPS coordinates in this list with the other two sources based on fuzzy-matching pre-processed facility name strings (e.g., removing non-Latin characters, removing facility type strings) using the Jaro-Winkler metric. An initial threshold score of 0.2 was used to identify potential matches, followed by a manual verification process to identify and reconcile false positive and false negative matches, as well as include facilities not reflected in the DHIS2 list. The final consolidated list used in this study consisted of N = 23,185 facilities with 79% GPS completion.

We estimated travel time using a cost distance algorithm that accounts for variation in travel speeds across differences in land uses, terrain, and barriers to transport, such as forests and water bodies. High-resolution spatial datasets representing these features were used to generate a travel friction surface, which was used to compute accumulated travel time to the nearest health facility in AccessMod version 5 [25]. While AccessMod is capable of producing travel times for both walking and motorized scenarios, we focused on walking time for this analysis for simplicity. Results were similar for both, and motorized results are available in the supplementary information (see Supplementary Table S1).

To derive population coverage statistics, we integrated travel time data with population distribution information from WorldPop [26]. At each gridded location in the country (both urban and rural, in this case), we extracted the population and the travel time from each surface. The population distribution was compared to the population distribution implied by the travel times extracted at the ECV survey locations.

### 2.4. Statistical Analysis

Descriptive statistics were provided to understand the sample characteristics. We used logistic regression to assess the relationship among geographic accessibility, caregiver attitude toward vaccination, and zero-dose vaccination status. We ran four nested models, successively adding complexity: 1. travel time only; 2. travel time and attitude; 3. travel time and attitude and covariates (maternal age, educational level of the household head, and the child’s birth order); and 4. travel time and attitude and covariates with an interaction term between travel time and attitudes. The interaction term tests whether geographic access affects vaccine outcomes differently depending on the level of caregiver attitudes toward vaccination. Travel time was discretized into the following categories: under 5 min, 5–10 min, 10–20 min, 20–40 min, 40–60 min, 60 or more minutes. In addition to the covariates mentioned above, the survey (ECV2021 or ECV2022) was included as a covariate to control for changes in time and due to differences in survey implementation. Ninety-five percent confidence intervals are reported for all statistical tests. All analyses were conducted using R 4.3.1. The code is available at https://github.com/rburstein-IDM/access_attitude (accessed on 30 May 2024).

## 3. Results

### Sample Description

The study sample included 80,313 12–23-month-old children living in rural settings. After removing observations with missing or faulty GPS coordinates (N = 10,828, 13%), we were left with 69,485 observations, 33,489 (48%) from ECV2021 and 35,996 (52%) from ECV2022. Of these 16,157 (23%) were zero-dose, 22,770 (33%) had at least some vaccination, and 30,558 (44%) had completed their vaccination series. Nearly half (47%, or 33,063) of respondents lived within 5 min of a facility, and 88% (N = 61,230) of households were within 60 min. The ECV2022 included a sample of slightly more distant rural households than did the ECV2021 sample. [See Table A1].

**Figure A1:**
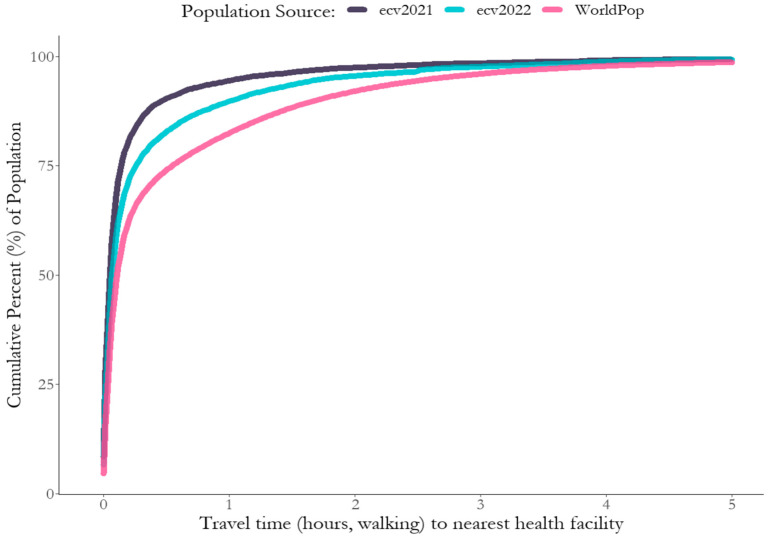
Travel time (walking) in hours to the nearest health facility. ECV2021 and ECV2022 samples were closer to health facilities than the population distribution implied by WorldPop. ECV2022 had a slightly more geographically distant sample, likely due to the addition of segmented sampling in that survey. Median travel time for the WorldPop population was 6.5 min compared to 3.5 in the full ECV sample, with 70% within 20 min (compared to 82% in ECV), and 88% within 60 min (92% in ECV).

**Figure A2:**
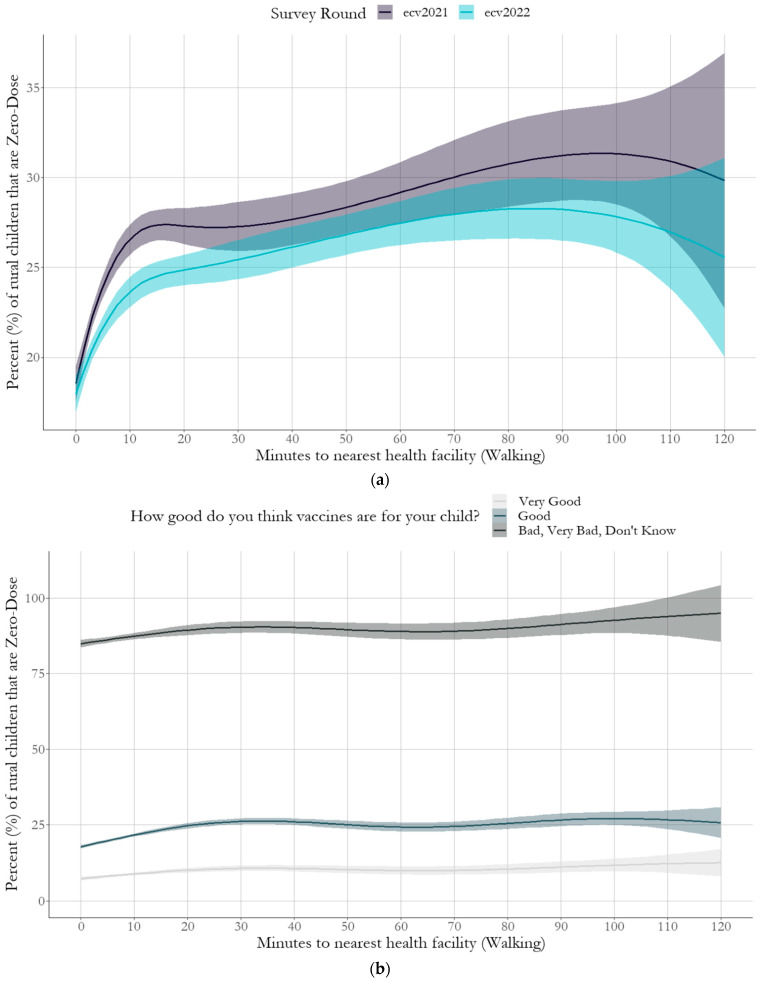
(**a**) Prevalence of zero-dose as a function of travel time (walking) in minutes to the nearest health facility, split by survey round. The lines represent smooths resulting from generalized additive models. (**b**) Prevalence of zero-dose as a function of travel time (walking) in minutes to the nearest health facility, split by parental attitude toward vaccines.

Figure A1 compares the population distribution walking time implied by the ECV surveys and WorldPop. Note again that here we included urban, as well as rural, households for a proper comparison. Relative to WorldPop, the ECV sample was nearer to health facilities. The median travel time for the WorldPop population was 6.5 min compared to 3.5 in the full ECV sample, with 70% within 20 min (compared to 82% in ECV), and 88% within 60 min (92% in ECV). Children who live further away from health facilities were more likely to be unvaccinated. Among children who live within 5 min of a health facility, 21% were zero-dose, while 26% of those living more than an hour from a facility were zero-dose (calculated from Table A1). In comparing travel time to the nearest facility and the prevalence of zero-dose, there was a steep increase in zero-dose prevalence within the first 10 min and a gradual increase in prevalence thereafter (Figure A2a). This relationship was similar in both survey rounds, with a slight shift downward in overall zero-dose prevalence in the 2022 survey.

Caregiver attitudes were strongly correlated with non-vaccination. Of the 9% of caregivers (N = 6285) who reported that vaccines are “Bad, Very Bad, or Dont Know”, 87% of their children were zero-dose. Only 8.6% of children were zero-dose among caregivers who rated vaccines as “Very Good” for their child. Among caregivers of children who were fully immunized, only 1% reported “Bad” or “Very Bad”, while 2% did among under-immunized children and 34% did among zero-dose children. However, within each level of parental attitude, there still appeared to be some effect of travel time (Figure A2b). Figure A2b emphasizes that across every time point in travel time, having a lower attitude status was the more important factor.

The modeled effect of caregiver attitude was much greater than the effect of travel time and was also not impacted much by the addition of the control variables. Unless otherwise noted, reported results are for the fully specified model (Model 3). The odds of being zero-dose for those living over an hour from a health facility relative to those living <5 min from a facility was 1.3 [95%CI: 1.2–1.4] (Table A2). There was a slight decrease from 40–60 min and 60+, but this difference was not significant. The effect of travel time was stable across the models, indicating that there was minimal confounding between the covariates and travel time (Table A2). In contrast, the odds ratio (OR) for “Very bad, bad, don’t know” relative to “Very good” was 69.3 [63.4–75.8]. Furthermore, the OR for “Good” in reference to “Very Good” was 2.8 [2.6–2.9]. Covariates, such as maternal age (40+ in reference to <18) and birth order (third or more in reference to first), were in the same order as the geographic access effect (ORs = 1.3 and 1.2 respectively). The effect of education (less than primary in reference to post-secondary) was greater than that of distance (OR: 2.0, 1.8–2.3). We found no significant effect of interaction between travel time and caregiver attitude or on the effect of survey year (see Supplementary Table S2).

## 4. Discussion

We linked estimates of travel time to the 2021 and 2022 ECV vaccination coverage survey to compare the relative effect of caregiver attitudes towards vaccinations and geographic accessibility to health facilities on child vaccination status. We found that caregiver attitudes toward childhood vaccination, a proxy for intent to vaccinate, is a many-fold stronger predictor of non-vaccination than geographic accessibility. Furthermore, both the ECV and WorldPop indicate that geographic accessibility is high in rural DRC, with 83% of the population living within an hour walk of a health facility.

In recent years, there has been a growing number of geospatial analyses on accessibility to healthcare in low- and middle-income countries [27,28,29] and the impacts of that on utilization [30,31,32,33,34,35,36,37] and health outcomes [38,39]. At the same time, it has been well understood that demand for services is a critical component of the uptake of health services, including vaccine services [40]. Our study contributes to this body of literature by comparing the relative influence of these two important determinants of utilization jointly. We believe this is the first time this comparison has been performed in DRC.

Our findings add to a growing body of evidence on geographic accessibility and caregiver attitudes to health services in DRC. A previous analysis of the ECV2021 on caregiver-reported reasons for non-vaccination found that 8% of zero-dose caregivers reported distance as a reason for not vaccinating their children, while 64% cited reasons related to people’s perceptions and feelings “Confidence in vaccine benefits” [22]. The present study strengthens this finding by confirming it through orthogonally measured variables, rather than self-reported reasons. In a study on antenatal care attendance in two provinces in DRC, Mafuta and Kayembe found that 83.5% of women reported that the distance to reach health facilities was less than 5 km and for 78.4% this distance took them less than an hour [41]. Our study also revealed that vaccine uptake was sensitive to short distances, with a steep increase in zero-dose prevalence from 0–10 min, and that in the model, being 20 min away was similar to being 60+ min away. Karra and colleagues found a similar pattern in their study of child mortality patterns in 21 low and middle-income countries [39].

Reaching zero-dose children is a growing global priority and is a centerpiece of Gavi’s 5.0 strategy [42], which is currently disbursing $500M to countries [43] as part of their goal to reduce the global number of zero-dose by 50% by 2030. To achieve this ambitious goal, immunization programs must progress from understanding who is unvaccinated to why caregivers are not taking their children to be vaccinated. This information is critical for making strategic programmatic choices under resource constraints. For example, based on the evidence presented in this paper, an intensive remote rural strategy is likely to reach fewer children than a near-facility demand-generation strategy. Of course, other information, such as cost data, and information about other determinants [44], such as supply chain readiness, and logistical considerations are critical factors as well.

The DRC government committed to strengthening routine immunization services under the Mashako Plan, launched in 2018. However, the plan in its current form does not address demand-related barriers to vaccination and primarily focuses on supply-side improvements, such as increasing the number of immunization sessions, supportive supervision, vaccination stocks, and cold chain improvement [11,45]. To fully address non-vaccination in the country, there will likely need to be an explicit focus on demand generation, for example by mobilizing the network of community health workers (relais communautaire, RECOs in DRC) for educational campaigns.

This paper motivates several areas for future study. First, given the importance of caregiver attitudes toward vaccination, a more thorough investigation of the intent to vaccinate is needed. In addition to caregiver attitudes toward vaccination, components like awareness, knowledge, agency, and community norms interact in complex ways to yield an intent to vaccinate [46,47]. Implementation of the WHO’s BeSD questionnaire in future surveys will be a welcome increase in the availability of such data [16]. Further studies should also aim to include a fuller set of determinants across intent to vaccinate, community access, and health facility readiness. For example, future studies may also include measures of health facility readiness to deliver vaccination services (e.g., WHO Service Availability and Readiness Assessment (SARA) immunization indicators) [48] and explore the relative prioritization of child immunization amongst the multiple priorities caregivers have to address possibly through a structural causal modeling framework, similar to work performed by Phillips et al. [44] Even in terms of access, the present analysis is relatively narrowly focused on geographic access, while access is a wider concept that encompasses the convenience and acceptability of services. As such, barriers, including wait times and caregiver costs (both direct and indirect), are not included here but may contribute significantly to the zero-dose status. Furthermore, variation in geographic accessibility due to weather, road conditions, and transportation should be further explored in subsequent studies (see limitations below). Finally, survey sampling in DRC is difficult as there is no recent census to draw on, and this may have impacted ECV sampling. Efforts underway by groups like GRID3 are making alternative geographic data sources available upon which to draw sampling frames for new approaches, such as gridded population survey sampling [49].

This paper has several limitations. First, the households sampled in ECV were nearer to health facilities than the population distribution in WorldPop. While we do not consider this a validation, as WorldPop does not necessarily represent ground truth, it indicates that there is some uncertainty around the true accessibility of the DRC population. The ECV2022 used an updated sampling methodology, segmenting each survey cluster into 16 areas spread across each enumeration unit, and randomly selecting six of them, which likely led to a slightly more remote population being selected (see Figure A1). For this reason, we primarily focus on our results on the modeled impact of distance, rather than reporting it as a representative estimate of distance to care. Second, there may be potential limitations in comparing the relative impact of self-reported attitudes and the objectively measured travel time. For example, while there may exist response bias (e.g., social desirability biases) where individuals do not accurately reflect their true attitudes towards vaccination, the same is not present for measures of travel time to vaccination. However, we note that the construct used to reflect attitudes has been well-validated in other contexts, mitigating some concern for such a bias in this context. Third, and relatedly, since attitudes toward vaccination were measured in the same survey as our outcome and travel time was measured independently, it is likely that travel time is measured with more error. This could lead to attenuation bias in the regression, meaning that the true effect of travel time was likely higher than our estimate. While this should be acknowledged, it is highly unlikely that the attenuation bias rises to the very large magnitude of the difference we observe in the effect of attitude versus access. The approach to estimating travel time may oversimplify the accessibility landscape by not accounting for variations in walking speeds, potential obstacles, or terrain conditions. Additionally, it may not fully capture the nuances of diverse transportation patterns within a population, particularly in areas where alternative modes of transport, such as bicycles or informal public transport, are common. As verification for our approach to estimating travel time, we found that parents who live further away are more likely to cite distance as a reason for non-vaccination (see Supplementary Figure S1). Finally, our operationalization of travel time does not fully account for travel time to all vaccination services. In DRC, some vaccination services are administered via outreach sessions, and we did not have enough information to include those in this analysis. Such strategies are meant to improve equity by reducing the cost for more remote populations to seek care. It is possible that the relatively weak impact of travel time that we observed in our analysis reflects the success of outreach strategies. A more detailed study of the impact of outreach services in DRC is needed.

## 5. Conclusions

Understanding the relative influence of barriers to vaccination is a critical step in developing effective strategies to reach zero-dose children. In the case of rural areas in DRC, attitudes toward vaccination are much more impactful than geographic accessibility. This evidence suggests that efforts to improve demand and address parental concerns will be a critical component of addressing the large zero-dose problem in DRC.

## Data Availability

The data underlying this study from the ECV surveys contain sensitive geographic information. Consequently, these datasets are not publicly available to ensure the privacy and confidentiality of the participants involved. Data access can be formally requested by contacting eric.mafuta@unikin.ac.cd. Travel time maps and health facility list will be made available as digital supplements.

## Supplementary Materials

The following supporting information can be downloaded at: https://www.mdpi.com/article/10.3390/vaccines12060617/s1, Figure S1: The proportion of caregivers who state that distance to care is a factor for non-vaccination increases from 5% next door to over 15% one hour away with distance to the nearest health facility; Table S1: The fully specified model (Model 3) using motorized travel time instead of walking travel time. Results are generally comparable to the walking model; Table S2: Model 4: fully specified model (Model 3), with additional interaction between travel time and parental attitude included. No consistent evidence of interaction was detected; Table S3: Model 3 sensitivity analysis, keeping in all observations with duplicate GPS coordinates. (N= 76,174). Results were nearly identical to the main model; Brief Description of ECV sampling.

## Appendix A

**Table A1:** Sample Characteristics.

	Fully-Immunized		Under-Immunized		Zero-Dose		Total	
	ECV 2021	ECV 2022	ECV 2021	ECV 2022	ECV 2021	ECV 2022	ECV 2021	ECV 2022
	(N = 14,227)	(N = 16,331)	(N = 11,251)	(N = 11,519)	(N = 8011)	(N = 8146)	(N = 33,489)	(N = 35,996)
Travel Time								
<5 min	7913 (55.6%)	7454 (45.6%)	5885 (52.3%)	4944 (42.9%)	3760 (46.9%)	3107 (38.1%)	17,558 (52.4%)	15,505 (43.1%)
5–10 min	2180 (15.3%)	2361 (14.5%)	1872 (16.6%)	1615 (14.0%)	1369 (17.1%)	1144 (14.0%)	5421 (16.2%)	5120 (14.2%)
10–20 min	1456 (10.2%)	1791 (11.0%)	1350 (12.0%)	1386 (12.0%)	990 (12.4%)	986 (12.1%)	3796 (11.3%)	4163 (11.6%)
20–40min	988 (6.9%)	1524 (9.3%)	881 (7.8%)	1248 (10.8%)	701 (8.8%)	981 (12.0%)	2570 (7.7%)	3753 (10.4%)
40–60min	463 (3.3%)	875 (5.4%)	392 (3.5%)	667 (5.8%)	391 (4.9%)	556 (6.8%)	1246 (3.7%)	2098 (5.8%)
60+ min	1227 (8.6%)	2326 (14.2%)	871 (7.7%)	1659 (14.4%)	800 (10.0%)	1372 (16.8%)	2898 (8.7%)	5357 (14.9%)
How good are vaccines?								
Very Good	5297 (37.2%)	7156 (43.8%)	2886 (25.7%)	3979 (34.5%)	732 (9.1%)	1096 (13.5%)	8915 (26.6%)	12231 (34.0%)
Good	8840 (62.1%)	9001 (55.1%)	8124 (72.2%)	7236 (62.8%)	4630 (57.8%)	4223 (51.8%)	21594 (64.5%)	20460 (56.8%)
Bad, Very Bad, Don’t Know	90 (0.6%)	174 (1.1%)	241 (2.1%)	304 (2.6%)	2649 (33.1%)	2827 (34.7%)	2980 (8.9%)	3305 (9.2%)
Mother’s Age								
<18	213 (1.5%)	324 (2.0%)	188 (1.7%)	249 (2.2%)	167 (2.1%)	171 (2.1%)	568 (1.7%)	744 (2.1%)
18–19	698 (4.9%)	1168 (7.2%)	690 (6.1%)	953 (8.3%)	525 (6.6%)	646 (7.9%)	1913 (5.7%)	2767 (7.7%)
20–24	4076 (28.6%)	4961 (30.4%)	3432 (30.5%)	3697 (32.1%)	2472 (30.9%)	2663 (32.7%)	9980 (29.8%)	11321 (31.5%)
25–29	3522 (24.8%)	3973 (24.3%)	2579 (22.9%)	2564 (22.3%)	1753 (21.9%)	1734 (21.3%)	7854 (23.5%)	8271 (23.0%)
30–39	4826 (33.9%)	4968 (30.4%)	3714 (33.0%)	3358 (29.2%)	2533 (31.6%)	2291 (28.1%)	11073 (33.1%)	10617 (29.5%)
>40	711 (5.0%)	659 (4.0%)	524 (4.7%)	528 (4.6%)	475 (5.9%)	480 (5.9%)	1710 (5.1%)	1667 (4.6%)
Missing	181 (1.3%)	278 (1.7%)	124 (1.1%)	170 (1.5%)	86(1.1%)	161 (2.0%)	391 (1.2%)	609 (1.7%)
Education Level of HH Head								
Tertiary	869 (6.1%)	1058 (6.5%)	418 (3.7%)	468 (4.1%)	182 (2.3%)	210 (2.6%)	1469 (4.4%)	1736 (4.8%)
Secondary	8295 (58.3%)	9842 (60.3%)	6224 (55.3%)	6511 (56.5%)	4136 (51.6%)	4359 (53.5%)	18655 (55.7%)	20712 (57.5%)
Primary	3776 (26.5%)	4112 (25.2%)	3500 (31.1%)	3312 (28.8%)	2713 (33.9%)	2600 (31.9%)	9989 (29.8%)	10024 (27.8%)
Less than primary	1099 (7.7%)	1063 (6.5%)	970 (8.6%)	1014 (8.8%)	847 (10.6%)	849 (10.4%)	2916 (8.7%)	2926 (8.1%)
Missing	188 (1.3%)	256 (1.6%)	139 (1.2%)	214 (1.9%)	133 (1.7%)	128 (1.6%)	460 (1.4%)	598(1.7%)
Birth Order								
1	6586 (46.3%)	8298 (50.8%)	5042 (44.8%)	5565 (48.3%)	3757 (46.9%)	4035 (49.5%)	15385 (45.9%)	17898 (49.7%)
2	6498 (45.7%)	6962 (42.6%)	5225 (46.4%)	5017 (43.6%)	3427 (42.8%)	3463 (42.5%)	15150 (45.2%)	15442 (42.9%)
3+	1143 (8.0%)	1071 (6.6%)	983 (8.7%)	937 (8.1%)	826 (10.3%)	648 (8.0%)	2952 (8.8%)	2656 (7.4%)
Missing	0 (0%)	0 (0%)	1 (0.0%)	0 (0%)	1 (0.0%)	0 (0%)	2 (0.0%)	0 (0%)

**Table A2:** Regression Results.

		Model 3: Fully-Specified (R-Squared = 0.30)			Model 2: Travel Time + Attitudes (R-Squared = 0.29)			Model 1: Travel Time (R-Squared = 0.01)		
Variable	Variable Level	OR	2.5% CI	97.5% CI	OR	2.5% CI	97.5% CI	OR	2.5% CI	97.5% CI
Intercept		0.1	0.0	0.1	0.1	0.1	0.1	0.3	0.3	0.3
Travel time	<5 min (Reference)									
	5–10 min	1.2	1.1	1.3	1.2	1.2	1.3	1.2	1.1	1.3
	10–20 min	1.3	1.2	1.4	1.3	1.2	1.4	1.3	1.2	1.3
	20–40 min	1.4	1.3	1.5	1.5	1.4	1.6	1.4	1.3	1.5
	40–60 min	1.4	1.3	1.6	1.4	1.3	1.6	1.5	1.4	1.6
	60+ min	1.3	1.2	1.4	1.3	1.3	1.4	1.4	1.3	1.4
Attitude	Very Good (Ref.)									
	Good	2.8	2.6	2.9	2.8	2.7	3.0			
	Bad/Very Bad/Don’t Know	69.3	63.4	75.8	71.7	65.7	78.3			
Maternal Age	Under-18 (Ref.)									
	18–19	1.0	0.8	1.2						
	20–24	1.0	0.9	1.2						
	25–29	0.9	0.8	1.0						
	30–39	0.9	0.8	1.1						
	40+	1.3	1.1	1.6						
HH Education	More than secondary (Ref.)									
	Secondary	1.7	1.5	12.0						
	Primary	2.0	1.8	2.3						
	Less than Primary	2.0	1.8	2.3						
Birth Order	First									
	Second	1.0	1.0	1.0						
	Third or more	1.2	1.2	1.3						
Survey Round	ECV2021 (Ref.)									
	ECV2022	0.9	0.9	1.0						
