# Supplementary material for: Zero-Dose Childhood Vaccination Status in Rural Democratic Republic of Congo: Quantifying the Relative Impact of Geographic Accessibility and Attitudes toward Vaccination
Source: Vaccines (Basel). 2024 Jun 4;12(6):617. doi: 10.3390/vaccines12060617 (PMC11209617; doi:10.3390/vaccines12060617)
Supplement: Supplementary file 1 [file vaccines-12-00617-s001.zip › vaccines-2971429-supplementary.pdf]

Supplementary information

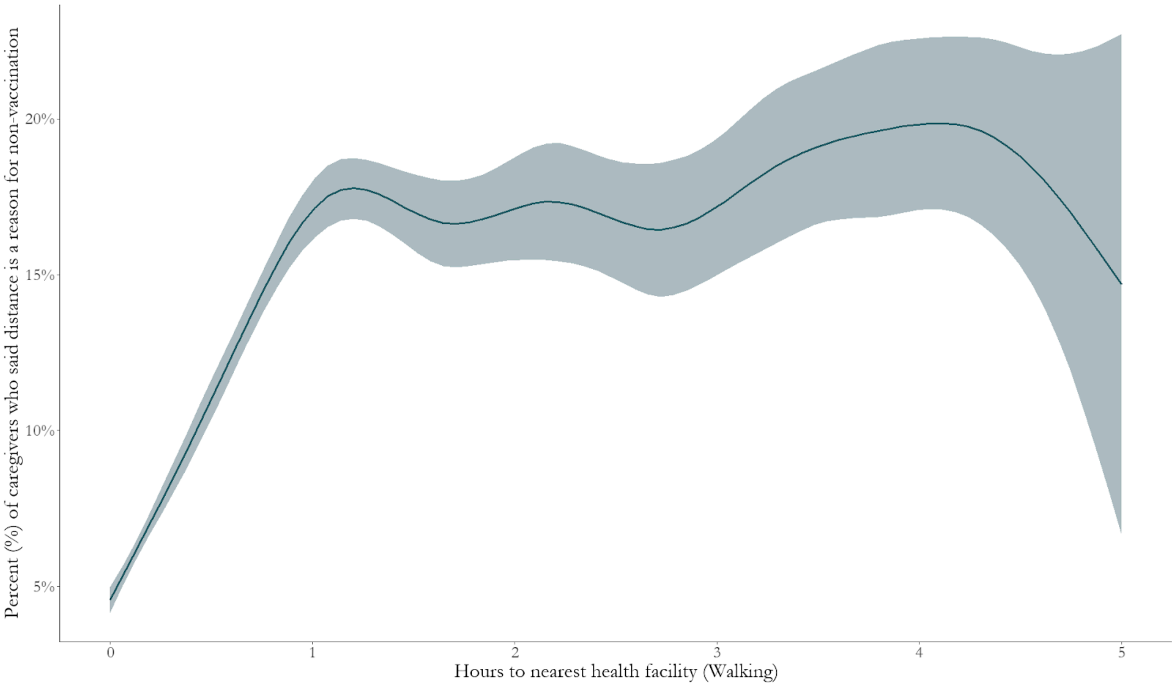

Supplementary Figure S1: The proportion of caregivers who state that distance to care is a factor for non-vaccination increases from 5% next door to over 15% one hour away with distance to the nearest health facility.

		Model 3 (motorized)		
Variable	Variable Level	OR	2.5% CI	97.5% CI
Intercept		0.05	0.04	0.06
Travel time	<5 minutes (Reference)			
	5 - 10 minutes	1.06	1	1.14
	10 - 20 minutes	1.13	1.05	1.22
	20 - 40 minutes	1.15	1.05	1.25
	40 - 60 minutes	1	0.88	1.14
	60+ minutes	1.21	1.11	1.31
Attitude	Very Good (Ref.)			

	Good	2.77	2.63	2.93
	Bad/Very Bad/Don't Know	69	63.1	75.44
Maternal Age	Under-18 (Ref.)			
	18-19	0.98	0.83	1.16
	20-24	0.99	0.86	1.16
	25-29	0.88	0.75	1.03
	30-39	0.92	0.79	1.07
	40+	1.31	1.11	1.56
HH Education	More than secondary (Ref.)			
	Secondary	1.76	1.55	1.98
	Primary	2.1	1.86	2.38
	Less than Primary	2.08	1.81	2.39
Birth Order	First			
	Second	1	0.96	1.05
	Third or more	1.25	1.16	1.35
Survey Round	ECV2021 (Ref.)			
	ECV2022	0.95	0.92	1

Supplementary Table S1: The fully specified model (Model 3) using motorized travel time instead of walking travel time. Results are generally comparable to the walking model.

		Model 4 (R^2= 0.30)		
Variable	Variable Level	OR	2.5% CI	97.5% CI
Intercept		0.05	0.04	0.06
Travel time	<5 minutes (Reference)			
	5 - 10 minutes	1.11	0.96	1.28
	10 - 20 minutes	1.42	1.22	1.65
	20 - 40 minutes	1.27	1.07	1.51
	40 - 60 minutes	1.46	1.17	1.83

	60+ minutes	1.24	1.06	1.46
Attitude	Very Good (Ref.)			
	Good	2.73	2.51	2.97
	Bad/Very Bad/Don’t Know	65.65	57.69	74.7
Maternal Age	Under-18 (Ref.)			
	18-19	0.98	0.83	1.16
	20-24	1	0.86	1.16
	25-29	0.88	0.76	1.03
	30-39	0.92	0.79	1.07
	40+	1.31	1.1	1.55
HH Education	More than secondary (Ref.)			
	Secondary	1.72	1.52	1.95
	Primary	2.04	1.8	2.31
	Less than Primary	2.01	1.75	2.31
Birth Order	First			
	Second	1	0.95	1.04
	Third or more	1.24	1.15	1.34
Survey Round	ECV2021 (Ref.)			
	ECV2022	0.94	0.9	0.98
Attitude (Good) * Travel time interaction. Attitude reference = Very Good	<5 minutes (Reference)			
	5 - 10 minutes	1.09	0.93	1.28
	10 - 20 minutes	0.88	0.75	1.05
	20 - 40 minutes	1.13	0.94	1.37
	40 - 60 minutes	0.99	0.77	1.27
	60+ minutes	1.04	0.87	1.25
Attitude (Bad, Very Bad, Don't Know) * Travel time interaction. Attitude reference = Very Good	<5 minutes (Reference)			
	5 - 10 minutes	1.43	1.08	1.88
	10 - 20 minutes	0.86	0.65	1.15

	20 - 40 minutes	1.35	0.95	1.91
	40 - 60 minutes	0.76	0.52	1.12
	60+ minutes	1.1	0.83	1.47

Supplementary Table S2: Model 4: fully specified model (Model 3), with additional interaction between travel time and parental attitude included. No consistent evidence of interaction was detected.

		Model 3 with duplicate GPS kept		
Variable	Variable Level	OR	2.5% CI	97.5% CI
Intercept		0.05	0.04	0.06
Travel time	<5 minutes (Reference)			
	5 - 10 minutes	1.21	1.14	1.28
	10 - 20 minutes	1.26	1.19	1.35
	20 - 40 minutes	1.42	1.32	1.51
	40 - 60 minutes	1.39	1.27	1.52
	60+ minutes	1.28	1.2	1.36
Attitude	Very Good (Ref.)			
	Good	2.77	2.63	2.92
	Bad/Very Bad/Don't Know	67.89	62.43	73.83
Maternal Age	Under-18 (Ref.)			
	18-19	0.93	0.8	1.09
	20-24	0.95	0.82	1.09
	25-29	0.84	0.72	0.96
	30-39	0.88	0.77	1.02
	40+	1.25	1.07	1.47
HH Education	More than secondary (Ref.)			
	Secondary	1.71	1.52	1.92
	Primary	2.03	1.81	2.28
	Less than Primary	2.01	1.76	2.28

Birth Order	First			
	Second	1.03	0.99	1.07
	Third or more	1.3	1.22	1.39
Survey Round	ECV2021 (Ref.)			
	ECV2022	0.97	0.93	1.01

Supplementary Table S3: Model 3 sensitivity analysis, keeping in all observations with duplicate GPS coordinates. (N= 76,174). Results were nearly identical to the main model.

Brief Description of ECV Sampling:

ECV samples are collected across the 26 provinces of the DRC, representing an intermediate level of the health system. These provinces are composed of secondary-level units, Health Zones (HZs), which constitute the operational level. Overall, the DRC's health system consists of 519 HZs, further subdivided into 8,504 Health Areas (HA). Data collection occurred in 511 HZs, while the remaining 8 HZs were excluded due to security concerns. Within each sampled HZ, 5 HAs were randomly selected from the list generated from DHIS2. Within each selected HA, 30% of villages or streets were chosen from the list provided by politico-administrative authorities. Subsequently, within each village/street, 34 households with a child meeting the criteria aged 6-23 were included in the study using a systematic random sampling method. Data collection for ECV2021 occurred in Feb-Apr 2022, and ECV2022 in Feb-Apr 2023. The sampling designs of both ECV rounds were similar, with a few modifications on ECV2202, where each selected HA was divided into 16 segments, with 6 of these segments avenues or villages were chosen. This approach aimed to ensure a certain degree of geographic dispersal within each HA and reduce bias resulting from field investigators' deliberate selection of locations.
